# Endothelial microparticles and vascular parameters in subjects with and without arterial hypertension and coronary artery disease

**DOI:** 10.1016/j.dib.2018.04.149

**Published:** 2018-05-08

**Authors:** Roberto Sansone, Maximilian Baaken, Patrick Horn, Dominik Schuler, Ralf Westenfeld, Nicolas Amabile, Malte Kelm, Christian Heiss

**Affiliations:** a Division of Cardiology, Pulmonology, and Vascular Medicine, Medical Faculty, University Duesseldorf, Duesseldorf, Germany; b CARID-Cardiovascular Research Institute Duesseldorf, Medical Faculty, University Duesseldorf, Duesseldorf, Germany; c Institut Mutualiste Montsouris, Paris, France; d University of Surrey, Faculty of Health and Medical Sciences, Guildford, UK; e Surrey and Sussex Healthcare NHS Trust, Redhill, UK

## Abstract

Endothelial microparticles (EMPs) are markers of endothelial injury and activation. The role of EMPs in arterial hypertension is not well understood and EMPs are increased both in arterial hypertension and coronary artery disease (CAD). The data presented here show EMPs as defined by CD31^+^/41^−^, CD62e^+^, and CD144^+^ surface markers and vascular hemodynamic parameters including office and central blood pressure, heart rate, aortic augmentation index, pulse wave velocity, flow-mediated dilation, nitroglycerin-mediated dilation, brachial artery diameter, hyperemic wall shear stress, and laser Doppler perfusion of the cutaneous microcirculation of normotensives and hypertensives with and without CAD.

## Specifications Table

**Table:** 

Subject area	*Biology*
More specific subject area	*Vascular pathophysiology*
Type of data	*Figures*
How data was acquired	*Flow-cytometry, sphygmomanometry, ultrasound, applanation tonometry, laser Doppler*
Data format	*Raw, analyzed*
Experimental factors	*Blood samples were taken along with vascular measurements in humans*
Experimental features	*Concentrations of endothelial microparticles in plasma and vascular hemodynamic parameters in normotensives and patients with arterial hypertension with and without stable coronary artery disease.*
Data source location	*Duesseldorf, Germany*
Data accessibility	*Data are presented in this article*

## Value of the data

• The data will aid the evaluation of endothelial microparticles (EMPs) as integrated markers of endothelial injury and activation.
• The data will help to understand the role of EMPs in the context of vascular changes in the pathophysiology of vascular disease showing changes due to in arterial hypertension alone and the interaction with coronary artery disease.
• The presented data will guide hypothesis-generation and design of further studies to understand EMPs in the context of development of vascular pathologies.

## 1 Data

**Fig. 1:**
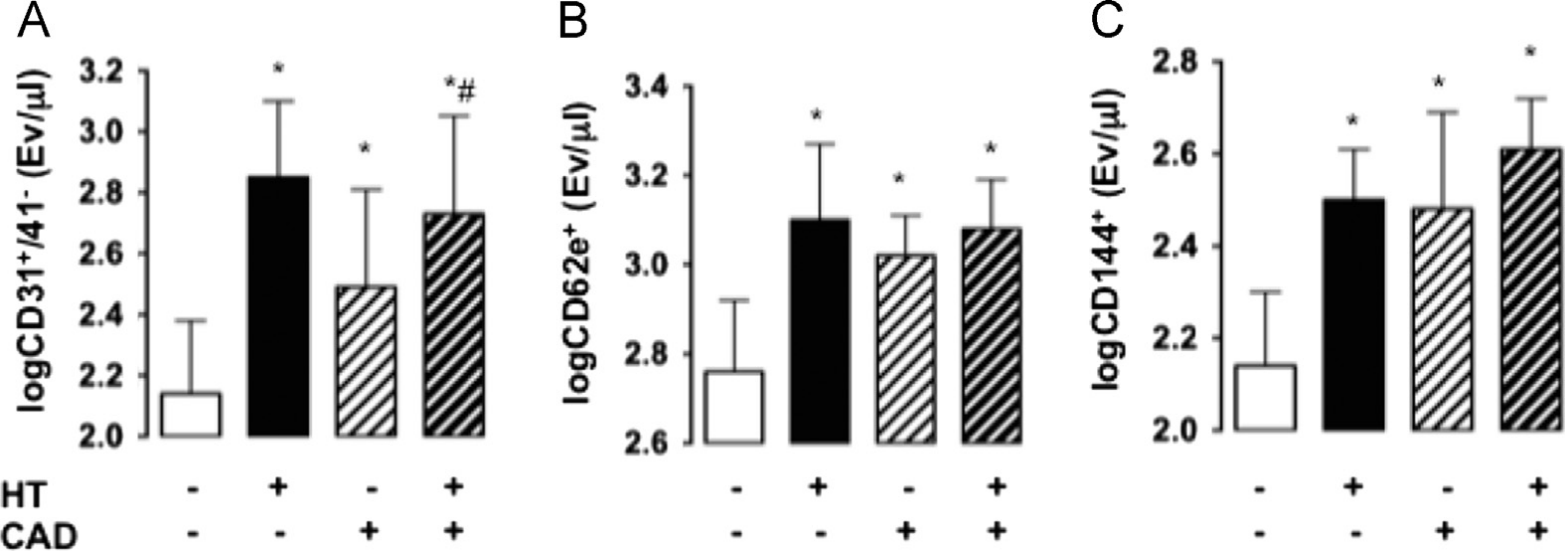
Endothelial microparticle (EMP) plasma concentration in normotensive patients (columns with white background) and patients with arterial hypertension (HT, columns with black background) with and without coronary artery disease (CAD, columns with diagonal lines). EMP subpopulations were discriminated by flow-cytometric analysis of platelet-free plasma according to the expression of membrane-specific antigens ([A] CD31^+^/CD41^−^, [B] CD62e^+^, and [C] CD144^+^; Values are mean and standard deviations of log10 transformed values; * *p* < 0.05 vs normotensive non-CAD, # *p* < 0.05 vs normotensive CAD.).

**Fig. 2:**
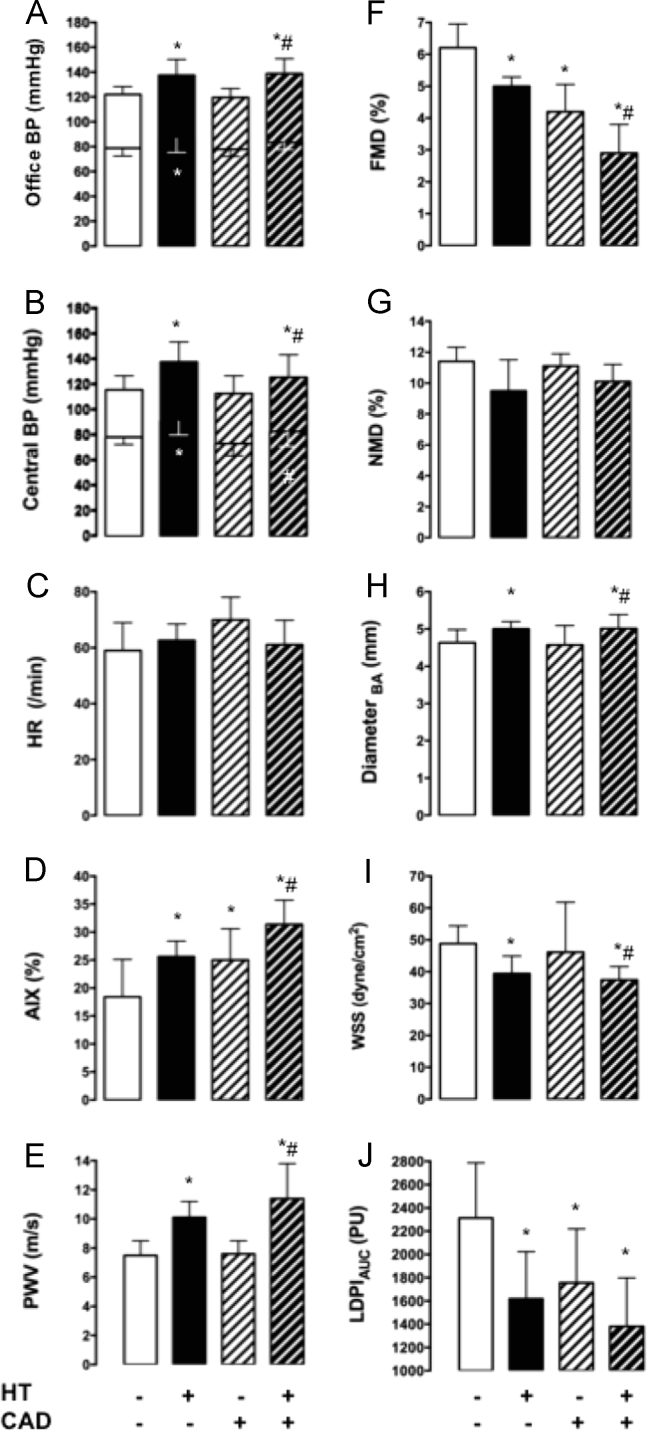
Hemodynamic characteristics of normotensive patients (columns with white background) and patients with arterial hypertension (HT, columns with black background) with and without coronary artery disease (CAD, columns with diagonal lines). (A) Office and (B) central blood pressure (BP), (C) heart rate (HR), (D) aortic augmentation index (AIX), (E) pulse wave velocity (PWV), (F) flow-mediated dilation (FMD), (G) nitroglycerin-mediated dilation (NMD), (H) diastolic diameter of the brachial artery (BA), (I) hyperemic wall shear stress (WSS), and (J) microvascular perfusion as measured by the hyperemic area under the curve (AUC) with laser Doppler perfusion imaging (LDPI). (Values are mean and standard deviations; * *p* < 0.05 vs normotensive non-CAD, # *p *< 0.05 vs normotensive CAD.).

The figures show endothelial microparticles (EMPs), as defined by CD31^+^/41^−^, CD62e^+^, and CD144^+^ surface markers (Fig. 1), and vascular hemodynamic parameters including office and central blood pressure, heart rate, aortic augmentation index, pulse wave velocity, flow-mediated dilation, nitroglycerin-mediated dilation, brachial artery diameter, hyperemic wall shear stress, and laser Doppler perfusion of the cutaneous microcirculation (Fig.2) of normotensive and hypertensive patients with and without CAD.

## 2 Experimental design, materials and methods

### 2.1 Design, inclusion and exclusion criteria

We investigated circulating EMPs along with functional and mechanical characteristics of the arterial system in 41 consecutive male subjects, 20 subjects without arterial hypertension and 21 subjects with arterial hypertension as defined by blood pressure > 140/90 mmHg or ongoing antihypertensive medication. The patients were recruited from the outpatient clinic. As the presence of CAD might also affect circulating EMP values, we aimed at including 50% patients with stable CAD. In all subjects, EMP subpopulations (CD31^+^/41^−^, CD62e^+^, CD144^+^) were analyzed by flow-cytometry according to the expression of surface antigens. Hemodynamics and endothelial function of the brachial artery were assessed as flow-mediated dilatation (FMD). Pulse wave analyses including central blood pressure, pulse wave velocity (PWV), and aortic augmentation index (AIX) were performed by tonometry. Exclusion criteria were manifest peripheral artery, or cerebrovascular disease, acute inflammation (CRP > 0.6 mg/dl), kidney failure (eGFR < 30 ml/min), malignancies, and arrhythmias (heart rhythm other than sinus).

### 2.2 Characterization of EMP subpopulations by flow cytometry

Citrated blood (6 ml) was drawn from the cubital vein and processed within 2 h. Platelet-rich plasma was obtained by centrifugation of whole blood at 300 *g* over 15 min at room temperature. Platelet-free plasma was obtained by 2 successive centrifugations of platelet-rich plasma at 10,000 *g* for 5 min at room temperature. Briefly, samples were incubated for 30 min with fluorochrome-labeled antibodies or matching isotype controls and analyzed in a Canto II flow cytometer (Beckton Dickinson, Heidelberg, Germany). Microbead standards (1.0 mμ) were used to define MPs as < 1 μm in diameter. The EMP subpopulations were defined as CD31^+^/CD41^−^, CD62e^+^, or CD144^+^ events. The total number of EMPs was quantified using flow-count calibrator beads (20 l).

### 2.3 Hemodynamic monitoring

The Task Force Monitor (CN-Systems, Graz, Austria) was used for continuous beat-to-beat assessment of cardiovascular variables, including stroke volume, BP, heart rate, and total peripheral resistance by impedance cardiography, which included ECG, phonocardiography, Finapres (Finapres-Medical-Systems, Amsterdam, Netherlands), and BP monitoring system (Dynamap, Tampa, USA) at the upper arm. We determined 24 h ambulatory BP measurements on the day before study days. Central BP was derived from peripheral pulse wave analysis (SphygmoCor^®^, AtCor-Medical, Australia).

### 2.4 Pulse wave analysis

Central blood pressure parameters including augmentation index (AIX) and pulse wave velocity (PWV) were measured by radial applanation tonometry using the SphygmoCor^®^ system in accordance with the recommendations of the expert consensus on arterial stiffness [1]. Via a proprietary generalized transfer function, the pressure waveform of the ascending aorta was synthesized. PWV was determined from tonometry measurements taken at the carotid and femoral artery.

### 2.5 Flow-mediated vasodilation (FMD)

Brachial artery (BA) FMD was measured by ultrasound (10 MHz transducer; Vivid I, GE) in combination with an automated analysis system (Brachial Analyzer, Medical Imaging Applications, Iowa City, IO) in a 21 °C temperature-controlled room after 15 min of supine rest [2]. A forearm blood-pressure cuff was placed distal to the cubital fossa and inflated to 250 mmHg for 5 min. Before cuff inflation, the patients were instructed to keep the forearm muscles relaxed during ischemia to avoid pain and all patients tolerated the cuff inflation well. Diameter and Doppler-flow velocity were measured at baseline and immediately after cuff deflation, at 20, 40, 60, and 80 s. FMD was expressed relative to baseline diameters as: (diameter_max_ − diameter_baseline_)/diameter_baseline_. Endothelium-independent nitroglycerin-mediated dilation (NMD) was measured at 4 min after 400 mg nitroglycerin (Nitrolingual forte, Pohl, Germany) in the brachial artery at the site of FMD measurements and calculated identically.

### 2.6 Wall shear stress (WSS)

WSS was assessed based on maximal brachial artery diameter and flow velocity measurements obtained during FMD measurements and was calculated as 8 * µ * mean flow velocity/mean diameter, where blood viscosity (µ) was assumed to be constant at 0.035 dyn/s cm^2^.

### 2.7 Microvascular function assessed by non-invasive laser Doppler imaging

All investigations were performed using a scanning laser Doppler perfusion imager (PeriScan PIM III System, Perimed, Sweden). The arm selected for measurements was immobilized to avoid moving artifacts using a vacuum pillow containing polyurethane beads, which molds to the shape of the arm (Germa, Sweden). The laser beam was positioned 15 cm above the forearm scanning a field of 200 mm^2^ (region of interest [ROI] = 8 · 8 pixels; three seconds per scan) on the volar site of the forearm. Microvascular reactivity was assessed during post-occlusive reactive hyperemia PORH. Following the baseline perfusion (one minute; 20 images), a blood pressure cuff located at the distal upper arm was inflated to suprasystolic pressure over 5 min. After the cuff release, the microvascular response on reactive hyperemia was recorded. Data acquisition and analysis were performed by LDPI Win Software (Perimed, Sweden) processing the perfusion as numerical values and color-coded-images [3]. The area under the curve (AUC) during reactive hyperemia was calculated with Prism 6 (GraphPad software, La Jolla, USA).

### 2.8 Statistical methods

Baseline characteristics of study subjects and results are described as average and standard deviation. Subgroup analyses between the 4 groups of patients were performed with one way ANOVA and Tukey post-hoc test. Normal distribution was confirmed by Kolmogorov–Smirnov test for all parameters except for subgroups of EMP data. As a conservative approach, we performed decadic logarithmic transformation of all EMPs concentrations. P values of less than 0.05 were regarded as statistically significant. All Analyses were performed with SPSS 23 (IBM Corp.).
